# Buprestid Beetles of Togo: Ecological, Sociocultural, and Nutritional Impacts of a High Quality Food Source

**DOI:** 10.3390/insects17030320

**Published:** 2026-03-16

**Authors:** Fègbawè Badanaro, Victor Benno Meyer-Rochow

**Affiliations:** 1 Laboratoire de Biochimie Appliquée à la Nutrition et à l’Alimentation, Faculté des Sciences, Université de Lomé, Lomé B.P. 1515, Togo; fbadanar@yahoo.fr; 2 Department of Ecology and Genetics, University of Oulu, FIN-90570 Oulu, Finland

**Keywords:** entomophagy, buprestid beetles, generational transition, nutrients, Togo

## Abstract

In the face of increasing pressure on agriculture and the effects of climate change, as well as the increasing popularity of Western foods, the enhancement of local food resources stands out as an essential strategy to strengthen food security and to slow down the erosion of traditions. Buprestid beetles are customarily consumed by some people in Togo, but these beetles remain poorly documented. This study explores the nutritional value of Buprestids consumed in Ecological Zone I of Togo. In total 630 individuals representing 7 ethnic groups from 14 localities, with 45 respondents in each, were interviewed. Buprestids were collected in the surveyed areas for identification, and specimens of *Sternocera interrupta* (Olivier, 1790) were specifically selected for biochemical analyses. Ash, amino and fatty acids, vitamins and fibre contents were determined using AOAC and AFNOR methods. Mineral content was determined by spectrophotometry; fatty acid composition by gas chromatography and amino acid composition by Biochrom 30+ analyzer. The results show that three species of Buprestids are still consumed in Togo, but that this practice is becoming increasingly rare among younger people. The decline can be attributed to a combination of ecological, sociocultural, and economic factors. The analysis of *S. interrupta* revealed the following composition (dry weight): 58.02 ± 0.18% protein, 6.63 ± 0.28% lipid, and 12.81 ± 0.49% fibre. From a micronutritional standpoint, the species is rich in vitamins and minerals, highlighting the need to conserve Buprestids for their nutritional value and role in food security amidst ecological transition, as well as their contribution to biodiversity.

## Simple Summary

With the growing impact of climate change and increasing pressure on agriculture as well as the increasing popularity of Western foods, it is essential to identify locally available traditional and sustainable food sources. In Togo, some people have customarily consumed insects, including buprestid beetles. However, this practice is becoming increasingly rare. To understand this trend, we surveyed 630 people across 14 villages in Ecological Zone I of Togo. We also analysed the nutritional composition of one specific species, *Sternocera interrupta*. The results revealed that this beetle is very rich in protein, fibre, vitamins, and minerals and contains essential fatty acids and amino acids as part of a healthy diet. These findings indicate that *S. interrupta* is a high-quality food source to be promoted. The consumption of this and related species of buprestid beetles could boost human health, slow down the replacement of traditional foods by Western products, while protecting biodiversity. This study highlights the importance of rediscovering, preserving and promoting traditional food resources, especially in the context of ecological transition.

## 1. Introduction

Climate plays a fundamental role in agricultural production, which is recognized as one of the human activities most sensitive to climate variability [1,2,3]. While the effects of climate on agricultural systems vary across regions of the world, they are particularly pronounced in developing countries, especially in tropical zones. In Africa, nearly 70% of the population relies on rain-fed agriculture, and around 80% of cultivated land is managed by smallholder family farms [4]. This high dependency makes rural communities extremely vulnerable to climate variability, directly affecting crop yields and threatening food security.

In West Africa, increased climate variability, linked to irregularities in the monsoon system, exposes rural populations to growing risks. This uncertainty is exacerbated by the poverty of households, which limits their capacity to cope with natural disasters [5]. Moreover, the lack of access to modern agricultural technologies like irrigation, mechanization, and quality inputs severely restricts the farmers’ adaptive capacity [6]. Fluctuations in rainfall lead to significant declines in agricultural productivity, directly threatening food security, and some major famines since the 1970s have largely been attributed to these climatic hazards [7]. Faced with challenges such as these, strengthening the resilience of the agricultural sector and anticipating climate shocks has become a strategic priority for regional development and food security. It has therefore been recommended to adapt agricultural production systems through agroecological approaches that integrate local practices (like the use of resilient crop varieties [8,9]) and make use of traditional knowledge. The integration of wild or semi-domesticated species into agricultural systems could improve productivity through their resistance to environmental stressors, while also contributing to dietary diversification and nutritional security. This is where edible insects come into play, even though many traditionally consumed food species remain poorly documented in terms of their diversity, abundance, and nutritional composition.

Among these marginalized food resources, buprestids, i.e., thermophilic beetles [10] that seek out burnt timber after bushfires to lay their eggs in [11], need to be mentioned. Buprestids (confirmed by a study on the functional anatomy of their eyes [12]), are mainly active during the day and in Ecological Zone I of Togo are observed during the short annual period between September and October. In this region, local populations, involving children, women, and men, collect the adult insects by hand or net from shrubs like *Acacia* spp. (Fabaceae) and use them as food; this traditional practice, however, is becoming less and less common these days. At the same time, the scientific knowledge of the diversity and nutritional value of edible buprestids, including their wood-boring, legless larvae known as flatheads, remains very limited with regard to Togo and Africa as a whole. In Northeast India, members of the Nyishi tribe [13] have been reported to boil or smoke *Sternocera* sp. adults, while the Chakma tribals [14] prefer to roast or fry these beetles.

Because of this dearth of knowledge in connection with edible buprestids specifically in Togo, the main objectives of this study were threefold: 1—to identify the species of buprestids most commonly consumed in Ecological Zone I of Togo and to explore their nutritional potential; 2—to gather information on the history and the practice to use buprestids as a food item in the study region, and 3—to analyse the chemical composition and the nutrient content of *S. interrupta*, the most frequently encountered buprestid species in the study area, in order to compare the data with those of other edible insect species elsewhere.

## 2. Materials and Methods

### 2.1. Study Area

**Figure 1:**
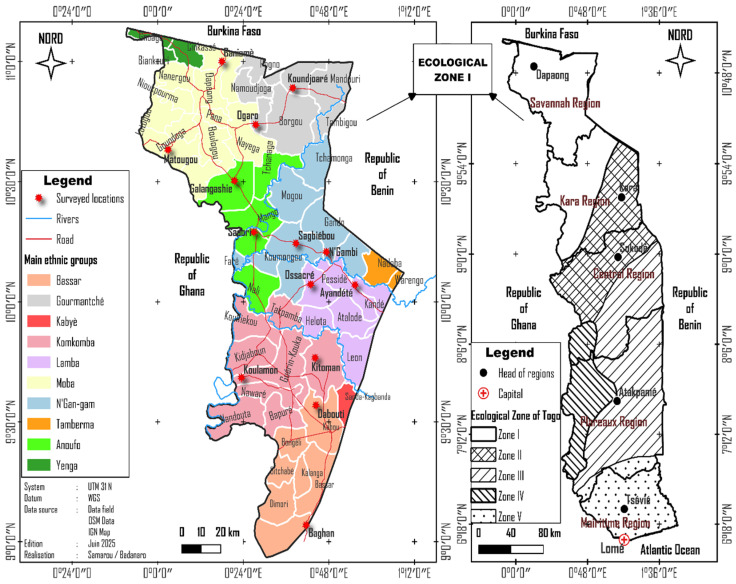
Surveyed Localities in Ecological Zone I of Togo.

Ecological Zone I extends across the Savannah Region and part of the Kara Region (Figure 1), covering an area of 8553 km^2^, or approximately 15% of the national territory. Its landscape consists of three major geomorphological units: the Dapaong and Bombouaka plateaus, the Birimian basement, and the plains of the Oti Valley [15]. The climate is of the Sudanian tropical type, characterized by two distinct seasons: a long dry season and a rainy season. Average annual rainfall is around 1000 mm, with significant year-to-year variation. Temperatures range between 20 and 40 °C during the dry season and between 22 and 34 °C during the rainy season, with an annual average of approximately 31.5 °C [16].

The hydrographic network is dominated by the Oti River, supplemented by the Kara, Mô, and Bina rivers and their tributaries. These rivers traverse a diverse vegetation landscape made up of savannas, dense dry forests, and gallery forests, which provide significant potential for non-timber forest products [17]. The region is largely covered by agroforestry parklands, whose products contribute significantly to improving the socioeconomic conditions of local populations [18]. In addition, sacred groves, traditional forms of biodiversity conservation, are commonly found near settlements [19]. A significant portion of the territory is included in environmental protection schemes, particularly within the Oti–Kéran–Mandouri protected area complex [20]. From a sociocultural perspective, the area is inhabited by several ethnic groups, including the Anoufo, Ngam-gam, Tamberma, Yanga, Lamba, Moba, Gourmantché, Konkomba, and Bassar [21]. Agriculture is the dominant activity, with the Savannah Region recognized as the country’s main livestock hub and a transit zone for transhumant cattle. The population derives its income primarily from farming and the collection of non-timber forest products, notably edible insects [16].

### 2.2. Data Collection

The methodological approach for data collection adopted in this study is based on ethnoentomological surveys using survey forms (Supplementary Materials) and field sampling of consumed buprestid beetles. Data were collected in 2022 during the period when buprestids are available, which is between September and October. This timeframe was selected based on previous ethnoentomological studies on edible insects in Togo [22]. Information was obtained through surveys conducted among people of different age groups (youth (5–30 years), adults (30–60 years), and elders (60–85 years)) in Ecological Zone I of Togo (Figure 1). These surveys focused on 7 major ethnic groups in the area to document the species of buprestids consumed. For each ethnic group, two localities were randomly selected, resulting in a total of 14 surveyed localities. In each locality, a sample of 45 individuals was interviewed, evenly distributed among the three age groups. A total of 630 individuals were surveyed. Responses were recorded using pre-designed data collection forms during individual semi-structured interviews.

To assist with species identification in the field, the interviewers were accompanied by local guides known for their expertise in edible insects. Buprestids were caught by sight using sweep nets. The beetles were then euthanized in jars containing an ethyl acetate pad and placed on layers of absorbent cotton prepared for this purpose. The host plants of the insects were carefully harvested with scissors. The collected specimens were transferred to the Applied Entomology Laboratory of the Faculty of Sciences at the University of Lomé, where they were identified to the species level using appropriate identification keys. Biochemical analyses focused on adults of *S. interrupta*, the species most frequently found in the study area. Specimens of this species were collected from three localities where the ethnoentomological surveys were conducted: Kitoman, N’Gambi, and Galangashie. The harvested insects were placed in a cooler, containing cold packs and ice cubes [23], before being transported for analysis to the Applied Biochemistry and Nutrition Laboratory of the Faculty of Sciences at the University of Lomé.

### 2.3. Biochemical Assays

Freshly collected adult specimens of *S. interrrupta* from each of the three localities were brought to the laboratory, weighed, and mixed to obtain an average sample. Such average samples were oven-dried at 40 °C until a constant weight was obtained. The beetles were then ground in a General Electric Interlabs Moulinex grinder. Ground samples of 20 g in weight were kept cool in a refrigerator for subsequent chemical analysis.

- Fibre content was determined using the Weende method [24].
- After acid hydrolysis followed by basic hydrolysis, the samples were dried at 150 °C for one hour and then incinerated at 550 °C for 6 h.
- Compositions of ashes (mineral substances), lipids, and proteins were determined according to methods of the Association of Official Analytical Chemists (see below).

The amounts of ash were obtained by incinerating the samples at 550 °C for 6 h while proteins were estimated by determining total nitrogen using the Kjeldahl method. After adding 0.2 g of selenium sulphate and 20 mL of sulfuric acid to 0.5 g of crushed insect, the mixture was heated until discoloration occurred. This discoloration indicates that all organic forms of nitrogen have been converted to ammonium sulphate. Ammonia was distilled by introducing the resulting mixture, along with methyl red and 75 mL of 40% sodium hydroxide, into the distillation apparatus. Heating the mixture releases ammonia from the ammonium sulphate in a basic medium, which is then distilled off. An Erlenmeyer flask, used to collect the distillate, contained 20 mL of 0.1 N sulfuric acid and Tashiro’s reagent as a color indicator. Once the pH paper indicated an acidic pH for the distilling solution, the distillation was stopped, and the excess acid was neutralized with a 0.1 N sodium hydroxide solution until the Tashiro reagent turned yellow-green. The percentage of nitrogen (% *N*) in the sample was calculated using the following formula:

(1) $$N=\frac{0.14\left(20-Volume\;of\mathrm{sodium}\;\mathrm{hydroxide}\;\mathrm{solution}\;\mathrm{used}\right)}{\mathrm{Mass}\;\mathrm{of}\;\mathrm{sample}}$$

The crude protein was calculated by multiplying percentage nitrogen by a constant factor of 6.25, i.e., % crude protein = % *N* × 6.25. The factor represents a nitrogen content of animal protein generally, but conversion factors for edible insect proteins (although variable) are somewhat lower and an average of 5.33 for edible insects has been suggested [25]. Therefore, our values are likely an overestimation of the protein content, but comparable to earlier insect studies that had also used a conversion factor of 6.25. The percentage of nitrogen-free extract (NFE) was calculated by subtracting the sum of crude protein, crude fat, crude fibre, and ash from 100. The calorific value (kJ/100 g) was estimated by multiplying the NFE and protein contents by 4, fat by 9, and fibre by 8, and then summing the resulting values [26].

Lipids were extracted with hexane using Soxhlet equipment (Auxilab, S.L. located in Beriáin, Spain), and the extracts were evaporated under vacuum at 35 °C using a Buchi R114 rotavapour (Flawil, Switzerland).

- Mineral content was determined using the Pauwels method [27].

Phosphorus content was obtained by colorimetry using the phosphovanado molybdate method, and absorbance was assessed using a colorimeter (Jenway model 6300, Livingston, UK). Other minerals (calcium, magnesium, potassium, sodium, iron, manganese, copper, and zinc) and heavy metals (cadmium, mercury, lead, arsenic, and nickel) were analyzed by atomic absorption spectrophotometry. The solubilization of the insect crushers was performed by acid attack on a sand bath, using two concentrated solutions: nitric acid and hydrogen peroxide. Indeed, one gram of each grind was introduced in a Teflon to which 1 mL of hydrogen peroxide and 8 mL of nitric acid were added. After stirring, the Teflons were heated on a sand bath for about 2 h at a temperature of about 150 °C. The recovery of the products obtained after heating was performed with 2 mL of distilled water. After cooling, the solution obtained after digestion was transferred to a 100 mL volumetric flask and supplemented with demineralized water. After homogenization, the solution was filtered through a Whatman paper. Thus, the filtrate was collected in a closed bottle. The determination of minerals was carried out from this filtrate by a flame atomic absorption spectrophotometer, Agilent 7500 ICP-MS cu UP 213 (Santa Clara, CA, USA), using the standard solutions. The real concentrations were determined with the following formula [27]:

(2) $$RC=\frac{CS\times DV}{M}$$

where *RC* is the real concentration, *CS* is the analyte concentration, *DV* is the dilution volume, and *M* is the mass of the test sample. To assess the nutritional quality of each species, the Ca/P, Ca/Mg, and Na/K ratios were calculated.

- The fatty acid composition of the lipids was obtained by gas chromatography, and the omega-6/omega-3 ratio was calculated.

An HP 6890 Series GC System gas chromatograph (Santa Clara, CA, USA) was used for the analysis. The instrument was equipped with a flame ionization detector and an HP-5 (cross-linked 5% ME siloxane) capillary column (length: 30 m; film thickness: 0.25 μm; internal diameter: 0.32 mm). The oven temperature was programmed to increase from −60 °C to +325 °C at a rate of 1 °C/min. The injector temperature was set at 275 °C, and the detector was set at 325 °C. The inlet nitrogen pressure, used as the carrier gas, varied from 6.90 to 47.6 kPa. The flow rate was maintained at 1 cm^3^/min, and the dead time was 1 min 15 s (hydrogen at 40 cm^3^/s). All fatty acids were analyzed in the samples.

Chromatographic profiling of fatty acids was carried out in two stages: sample preparation and determination by gas chromatography [28]. Sample preparation was conducted in accordance with ISO 5509 [29]. Five grams of fat were placed in a flat-bottomed flask, and 2.5 g of liquid sodium hydroxide (NaOH 1N) were added. A few grains of pumice stone were included to prevent excessive crackling. A cooler was fitted to the flask, and the mixture was heated under reflux over low heat until a crust formed. Slowly, 20 mL of 20% (*v*/*v*) sulfuric acid was added, and the mixture was heated until a translucent solution appeared. Next, 80 mL of the methanol–sulfuric acid mixture was added to the translucent solution, and the mixture was boiled under reflux for 2 h.

After 2 h of reflux boiling, the esterified fat was decanted into a separating funnel until the non-esterified phase was completely removed. A volume of 0.5 mL of the esterified sample was mixed with 2.5 mL of hexane, then decanted to obtain a solution of methyl esters. Gas chromatographic determination was carried out in accordance with ISO 5508 [28] by injecting one microliter (1 µL) of the methyl ester solution obtained. The peaks representing the methyl esters were identified using reference standards (methyl esters) by comparing the retention times of each peak in the chromatogram with those obtained for the standards.

The percentages of saturated and unsaturated fatty acids (monounsaturated and polyunsaturated) lipids were obtained by summing the contents of the fatty acids concerned. The omega-6 fatty acid found in the lipids of the insect species studied is linoleic acid, and the omega-3 fatty acid found is α-linolenic acid. The omega-6/omega-3 ratio was calculated based on the levels of these fatty acids in the samples.

- Amino acid composition was determined using a Biochrom 30+ amino acid analyser, following the method described by Fountoulakis and Lahm [30].

Fifteen (15) mg samples of the insect were weighed for extraction. A sample was transferred into a hydrolysis tube, to which 50 µL of norleucine (internal standard, 25 µmol/mL) and 450 µL of 4 N methane sulphonic acid were added. After degassing, the tube was sealed and placed in a hydrolysis apparatus at 150 °C for 120 min.

Following hydrolysis, the tubes were removed and allowed to cool at room temperature for 5 min. To stop the reaction, 450 µL of 4 N NaOH was added to the hydrolysate. The reaction mixture was then transferred to a 5 mL volumetric flask using a Pasteur pipette. The hydrolysis tube was rinsed three times with sodium citrate buffer (pH 2.2), and the rinse solutions were added to the flask. The final volume was adjusted to 5 mL with the same buffer.

The resulting solution was filtered through a Sartorius 0.45 µm membrane filter. The filtrate, containing a mixture of amino acids, was introduced into a cation exchange resin column via a refrigerated autosampler. Amino acid separation was achieved using Biochrom buffer solutions of varying pHs, combined with a temperature gradient controlled by a Peltier-effect oven.

Amino acid ratios were calculated using the reference source WHO/FAO/UN [31]. Amino acid ratios (*Raa*), in percent, were calculated according to the following formula:

(3) $$AAI=\frac{Content\;of\;an\;essential\;amino\;acid\;in\;the\;sample}{Favorable\;content\;of\;the\;homologous\;amino\;acid\;according\;to\frac{\frac{WHO}{FAO}}{UN}\left(1985\right)}\times 100$$

Amino acid number was then deducted for *S. interrrupta*. Indeed, the amino acid number of a species is the minimum value of the *Raa* of the species.

- Vitamins were assayed using AOAC [32] methods.

Vitamins in the various samples were determined by colorimetry. Optical density was measured using a Jenway model 6300 colorimeter. Calibration curves were obtained from the preparation of a range of solutions of the corresponding vitamin molecule. The samplings were prepared as follows:

Retinol (A): One gram of the sample was placed into a 250 mL flask. After adding 5 mL of pyrogallol solution, 35 mL of ethanol, and 10 mL of potassium hydroxide solution, the mixture was heated for 30 min at 70–80 °C under a reflux condenser, then allowed to cool under a stream of water. After cooling, 40 mL of distilled water and 100 mL of petroleum ether were added. Extraction was performed by stirring for 3 min. The mixture was then left to settle, and the upper phase was transferred to a separating funnel. The ethereal phase was washed to neutrality with three 50 mL portions of water and filtered through filter paper. A 5 mL sample of the ethereal phase was transferred into a 50 mL flask and diluted with petroleum ether. The retinol concentration of this solution was determined by measuring its optical density at 325 nm.

Thiamine (B1): The thiamine content of the samples was determined by adding 50 mL of 0.1 N sulfuric acid to one gram of each sample in a 100 mL volumetric flask. The mixture was heated in a water bath at 100 °C for 30 min, with frequent stirring. Five milliliters of 2.5 N sodium acetate solution were added to the contents, and the mixture was left to cool. After cooling, the flask was capped and placed in a water bath at 45–50 °C for 2 h. The resulting solution was made up to 100 mL with distilled water and filtered through filter paper. A 10 mL volume of the filtrate was transferred and mixed with 5 mL of potassium chloride solution. Absorbance was measured at a wavelength of 285 nm.

Riboflavin (B2): One gram of each sample was weighed into a 250 mL volumetric flask. To this, 5 mL of 0.1 N sulfuric acid and 5 mL of dichloroethane were added, followed by 90 mL of distilled water. The mixture was stirred and heated on a sand bath for 30 min to extract the riboflavin. Afterward, the mixture was cooled and made up to 250 mL with distilled water, then filtered through filter paper. A 2 mL volume of the filtrate was transferred into another 250 mL volumetric flask and topped up with distilled water. The riboflavin concentration of the solution was determined by measuring its absorbance at 460 nm.

Niacin (B3): Five grams of the sample were extracted with 50 mL of distilled water. Extraction was performed by repeated stirring for 30 min. The mixture was then left to settle, and the upper phase was recovered and filtered. This operation was repeated three times with the same amount of distilled water (100 mL). Five milliliters of the combined filtrates were transferred into a 100 mL volumetric flask and topped up with distilled water. The absorbance of the resulting colored solution, measured at a wavelength of 385 nm, was used to determine the nicotinic acid content of the sample.

Tocopherol (E): One gram of the sample was weighed and placed in a 250 mL flat-bottomed flask. A solution of 10 mL of ethanol and 20 mL of 1 N sulfuric acid was added. The flask was wrapped in aluminum foil and heated under reflux for 45 min. The resulting solution was cooled for 5 min, followed by the addition of 50 mL of distilled water, and transferred to an aluminum foil-covered separating funnel. The unsaponifiable matter in the mixture was extracted five times with 50 mL of dimethyl ether each time. The combined extract was washed with 1 N sulfuric acid solution and dried over anhydrous sodium sulfate. The evaporated extract was immediately dissolved in 15 mL of ethanol, 1 mL of concentrated sulfuric acid, and 1 mL of concentrated nitric acid. The resulting solution was placed in a water bath at 90 °C for 30 min. After cooling, the tocopherol content of the extract was measured by ultraviolet absorption at 470 nm.

### 2.4. Statistical Analyses

All statistical analyses were performed using SPSS (Statistical Package for the Social Sciences) version 27. To assess the existence of a statistically significant association between generations, ethnic groups, and the consumption of Buprestids, a chi-square test of independence was conducted. The objective was to determine whether consumption behaviors regarding Buprestids varied according to generational (youth, adults, elders) and/or ethnic group membership.

For the chemical analyses, all tests were conducted in triplicate. Means were calculated based on the three replicates and were reported along with their corresponding standard deviations (SDs) to reflect measurement variability.

## 3. Results

### 3.1. Diversity of Buprestid Beetles Consumed in Togo

Ethnoentomological investigations conducted in Ecological Zone I of Togo identified three edible species belonging to the family Buprestidae, commonly known as jewel beetles. These beetles are traditionally consumed by the various ethnic groups of the region (Table 1). The edible species comprise two genera, *Sternocera* and *Steraspis*, which are locally recognized for their nutritional value and are subject to seasonal harvesting. However, their availability, according to information from the surveyed populations, appears to have been declining in the last ten years if not longer.

### 3.2. Physiological and Socioeconomic Impacts of Buprestid Consumption in Togo

The Chi^2^ test indicated a significant generational difference in Buprestid consumption (χ^2^ = 108.077; *p* < 0.001), while no significant variation was observed across ethnic groups (χ^2^ = 3.417; *p* = 0.755) (Table 2). Consumption of buprestids tended to decline among younger generations compared to older ones. Additionally, no significant interaction was found between generation and ethnic group in relation to consumption patterns (χ^2^ = 4.882; *p* = 0.962).

### 3.3. Factors Behind the Decline in Buprestid Consumption

**Figure 2:**
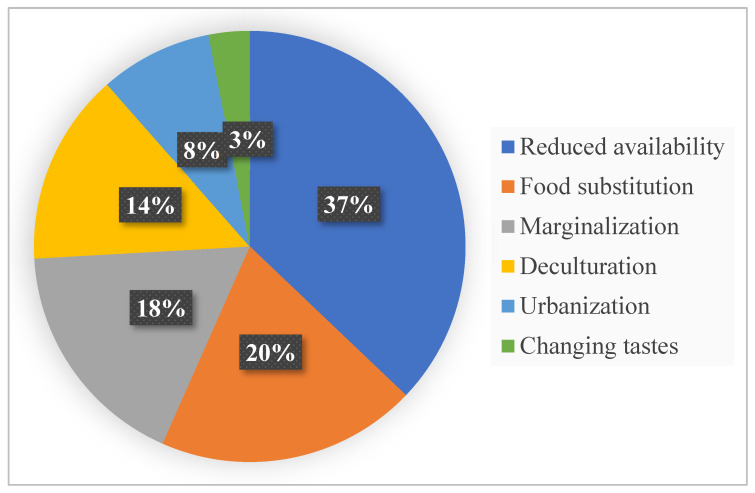
Reported reasons for the decrease in buprestid consumption.

Among the factors mentioned by respondents who consume buprestids to explain the decline in their consumption, reduced availability (37%) was the primary factor, followed by food substitution (20%) (Figure 2). Marginalization through a lack of opportunities (18%) and deculturation expressed as an alienation from traditions (14%) additionally play a significant role. Other factors, such as urbanization (8%) and changing tastes (3%), were also cited, although to a lesser extent.

### 3.4. Chemical Composition of S. interrrupta

#### 3.4.1. Proximate Composition and Energy Value

Table 3 shows the average composition of the *S. interrupta* samples. According to this table, 100 g dry weight (DW) of *S. interrupta* contained 58.02 ± 0.18 g of protein, 6.63 ± 0.28 g of fat, 12.81 ± 0.49 g of fibre, and 9.48 ± 0.45 g of ash. The protein content could be an overestimation (see Section 2.3) and fibre, as is the case with most other edible insects, would largely be the portion of the chitin in the exoskeleton.

#### 3.4.2. Mineral Composition

The mineral composition of *S. interrupta* shows that it is rich in macroelements like calcium (198.09 ± 1.82 mg/100 g), magnesium (98.82 ± 0.06 mg/100 g), phosphorus (183.28 ± 2.76 mg/100 g), potassium (635.56 ± 1.48 mg/100 g), and sodium (22.11 ± 0.17 mg/100 g), as well as in trace elements like iron (11.86 ± 0.02 mg/100 g), copper (2.84 ± 0.11 mg/100 g), zinc (11.49 ± 0.17 mg/100 g), and manganese (5.71 ± 0.07 mg/100 g) (Table 4). Compared with the mineral contents of the termite *Odontotermes* sp. (Termitidae) and the ant *Oecophylla smaragdina* (Fabricius, 1775) (Formicidae) [33], *S. interrupta* is particularly rich in calcium, similar to the flower scarab *Protaetia brevitarsus* (Lewis, 1879) [34]. Moreover, the consumption of 100 g of *S. interrupta* covers the entire recommended daily intake (RDI) of the elements iron, zinc, copper, and manganese [35]. The species can be said to be a valuable source of calcium, magnesium, and phosphorus, although it is low in sodium. Sodium/potassium, calcium/phosphorus, and calcium/magnesium ratios for *S. interrrupta* are 0.03, 1.08, and 2.00 respectively (Table 5).

Table 5 shows that the sodium/potassium, calcium/phosphorus, and calcium/magnesium ratios for *S. interrrupta* are 0.03, 1.08, and 2, respectively.

#### 3.4.3. Vitamin Composition

The vitamin contents of the studied species vary depending on the compounds (Table 6) per 100 g of product: retinol (0.02 ± 0.0 mg), thiamine (1.28 ± 0.15 mg), riboflavin (1.43 ± 0.05 mg), niacin (7.14 ± 0.02 mg), and tocopherol (4.10 ± 0.08 mg). Among the analyzed vitamins, 100 g of *S. interrupta* is sufficient to meet the recommended dietary intakes for an adult in terms of riboflavin (119% of the RDI) and thiamine (107% of the RDI). The species partially contributes to the requirements for niacin (51% of the RDI) and tocopherol (34%). However, the retinol content (0.02 ± 0.0 mg) remains very low, representing only 2.9% of the recommended value (0.7 mg/100 g).

#### 3.4.4. Amino Acid Composition

The amino acid composition of *S. interrupta* is presented in Table 7. Among the 20 major amino acids that make up proteins and are encoded in the human genome, 18 were identified in *S. interrupta.* The species contains the following essential (in italics) and semi-essential as well as non-essential amino acids.

Amino acid availability from pork, beef, veal, chicken, eggs, soybean, lentils, kidney beans, long beans, and cowpeas was compared with that of larvae, pupae and adult honey bees [36], the termite *Odontotermes* sp., and the ant *O. smaragdina* [33].

The amino acid index of this insect is 96%, which is slightly below the 100% reference value for high-quality protein (Table 8). An amino acid index below 100 indicates that the concentration of at least one indispensable amino acid is limiting. Tryptophan is the only limiting amino acid in *S. interrupta*. The values of the other essential amino acids found in this buprestid exceed the recommendations of the WHO, FAO, and UN [31].

#### 3.4.5. Lipid Characteristics

The results of the chemical screening conducted on the lipids of *S. interrupta* are shown in Table 9. The lipids of this species contain saturated fatty acids like lauric acid: 1.03 ± 0.02%, stearic acid: 9.12 ± 0.02%, and isopalmitic acid: 34.09 ± 0.01%. The total saturated fatty acid content in this species is 44.24 ± 0.03% (Table 9). A monounsaturated fatty acid in this species, as in many other insects, is oleic acid: 48.69 ± 0.02%. The polyunsaturated fatty acids found include linoleic acid: 3.84 ± 0.00%, and α-linolenic acid: 0.96 ± 0.01%. The total proportion of unsaturated fatty acids in the insect is 54.45 ± 0.83%. The omega-6/omega-3 ratio of the fatty acids in *S. interrupta* is 4.

## 4. Discussion

### 4.1. Species Diversity of Buprestids Consumed in Togo

It was reported from Northeast India that buprestid species of the genus *Sternocera* were consumed as food in boiled or smoked form by members of the Nyishi tribe [13] and roasted or fried by members of the Chakma [14]. Three species of buprestids are reported (this paper) to be consumed in Ecological Zone I of Togo: *Sternocera castanea*, *S. interrupta*, and *Steraspis squamosa*. The appreciation of the three edible species in the studied area, including those closely related to the ones consumed in India, reflects specific local knowledge of edible insects as well as a culturally based food selectivity. The buprestid species consumed in Ecological Zone I of Togo bear the same vernacular name within each of the surveyed ethnic communities. For example, they are called “*namonfal*” in Bassar, “*anatangala*” in Gourmatché, and “*linamimiè*” in Konkomba. In fact, very often, when multiple insect species are consumed together, they are collectively referred to by a single vernacular name [23,37]. This linguistic trend reflects ancient traditional knowledge about the species’ habits and behaviour [38]. The fact that these species belong to two distinct genera indicates that the consumption of buprestids is not limited to a single taxonomic lineage but likely relies on practical or organoleptic criteria (such as size, taste, texture, and seasonal availability). The limited diversity observed (three species) could also reflect a loss of local biodiversity. It underscores the need to preserve the natural habitats where these species live, as their disappearance would result in the loss of a potential nutritional resource. This finding calls for better documentation and promotion of local knowledge on entomophagy, as other edible species may exist but remain little known, unexploited, or forgotten by the younger generation.

### 4.2. Ecological and Socioeconomic Impacts of Buprestid Consumption in Togo

Buprestid consumption varies significantly depending on the age of the consumers, but not with the ethnic group. Moreover, the lack of interaction between these two factors indicates that the decline in consumption among younger generations occurs independently of their ethnic background. The lack of a significant difference in buprestid consumption among ethnic groups in Ecological Zone I of Togo may be explained by their shared location within the same phytogeographical zone. As a result, the vegetation formations, particularly the host plants of buprestids, are similar across the different ethnic groups. In general, according to Morris et al. [39], insect consumption has declined significantly over the years, especially among young Africans. According to respondents, the decrease in buprestid consumption among younger generations compared with older ones is explained by a combination of ecological (reduced availability), cultural (marginalization, deculturation, changing tastes and preferences), social (urbanization), and economic (food substitution, costs) factors. This same observation has been reported by several authors [40,41], and not just for Africa [42]. However, reduced availability (37%) is the most significant factor influencing buprestid consumption in Togo. This suggests that the increasing scarcity of buprestids in the environment is a major constraint to entomophagy in Togo, and a program to rear and breed *S. interrupta* might be a promising approach. The reduction may be due to environmental pollution, deforestation, unavailable burnt trees to deposit their eggs on, excessive pesticide use, climate change, and overharvesting of insect populations. Easier access to other protein sources (meat, fish, processed foods) accounts for 20% of the responses and undoubtedly has affected and reduced interest in buprestids as a food item among the younger generation.

The decline is also linked to the marginalization of insect consumption, generally (18%). Indeed, the lack of official recognition of this practice has led to stigmatization. Traditional foods are increasingly associated with poverty or rural lifestyles [43], and consuming insects was often (and sometimes still is) seen by Westerners as ‘uncivilized’ [44], so that this social stigma drives younger generations to abandon ancestral diets. Other closely related factors include deculturation (14%), characterized by the lack of intergenerational transmission of traditional food knowledge and practices, and urbanization (8%), which reflects the loss of connection with nature and traditional lifestyles. Changing tastes (3%), influenced by globalization, were also cited as one of the causes for this decline. However, the low importance attributed to changing tastes in the decline of buprestid consumption suggests that most consumers have not lost their taste for or their interest in buprestids as a food item.

### 4.3. Biochemical Analyses of S. interrupta

The ash resulting from the incineration of *S. interrupta* was used to quantify several essential minerals contained in this insect. The levels obtained for these minerals confirm that this buprestid species alone provides consumers with a sufficient amount of minerals. Compared with that of other edible insects [45], the mineral content is relatively high for the studied buprestid (9.48%) and almost identical to that of *Gryllus bimaculatus* (De Geer, 1773) (Orthoptera: Gryllidae) adults [34]. Sodium and potassium play a regulatory role in the human body’s water balance and help maintain acid-base equilibrium, and potassium was especially present in significant quantities in *S. interrupta*. Considering the Na/K ratio being as low as 0.03, the consumption of *S. interrupta* may have a positive effect in preventing cardiovascular diseases [46]. Calcium and phosphorus are essential for bone growth. Calcium deficiency leads to growth retardation, rickets in children, muscle spasms in adults, and osteoporosis in the elderly. Since intestinal absorption of calcium and bone mineralization are enhanced when the Ca/P ratio is balanced [47], as is the case with *S. interrupta*, the intake of these elements from this insect has to be seen as beneficial.

The calcium/magnesium ratio in *S. interrupta* is close to two, as it is in the honey bee *Apis mellifera* Linnaeus, 1758 (Apidae) [36]. A ratio such as this is likely to reduce the risk of osteoporosis [48]. Trace elements that support proper bodily function were also found in *S. interrupta*, including iron, zinc, copper, and manganese, and are basically similar to those reported from other edible insects. The mineral composition of *S. interrupta* reveals a promising nutritional profile, particularly in its contribution to the intake of essential trace elements. The data show that 100 g of dried *S. interrupta* provides substantial proportions of the recommended dietary intakes (RDI) for several minerals as established by ANSES [35], highlighting its potential as a complementary food source in human diets.

In addition to minerals, *S. interrupta* also contains micronutrients in the form of vitamins. In fact, *S. interrupta* contains appreciable amounts of all the key vitamins, except for retinol. It has significant levels of thiamine, riboflavin, niacin, and tocopherol, making it a nutritionally high-quality species. The studied species stands out particularly for its richness in vitamins, meeting or even exceeding the recommended daily intake of 100 g. These vitamins serve various functions in the body: vitamins A and E are antioxidants, while B vitamins function as coenzymes [49]. Compared with values reported for meat products, insects (and this holds true for buprestids, too) are generally known to be rich in various vitamins and minerals [50].

Based on comparisons with insects in which the protein content was determined as in this paper, *S. interrupta* can be regarded an important source of animal protein (58.02 ± 0.18%). However, this value is slightly lower than that of adult Orthoptera such as *G. bimaculatus* [34,51], *Brachytrupes orientalis* (Fabricius, 1775) (Gryllidae), and *Chondacris rosea* (De Geer, 1773) (Acrididae) [52], although it is higher than the protein content of the edible larvae of the beetles *Allomyrina dichotoma* (Linnaeus, 1771) (Scarabaeidae), *Protaetia brevitarsis* (Lewis, 1879) (Scarabaeidae), and *Tenebrio molitor* Linnaeus, 1758 (Tenebrionidae [33]. The quality of proteins in food is determined by their essential amino acid content. All essential amino acids were present in the protein fraction of *S. interrupta*. A comparison of the amino acid composition of this insect with the WHO/FAO/UN reference pattern [31] shows that the levels of almost all essential amino acids exceed the standard. As with other insects, tryptophan is the only limiting amino acid for *S. interrupta*, with an amino acid score of 96%. However, tryptophan levels are more difficult than other amino acids to determine accurately. Total tryptophan and sulphur-containing amino acids (cysteine and methionine) are difficult to measure when hydrolyzed with hydrochloric acid. In acidic conditions, tryptophan degrades, but may be measured in alkaline conditions. The high overall protein content of *S. interrupta*, however, suggests its potential in combating protein deficiency in developing countries. This insect that is still readily available and affordable for indigenous populations in the areas where it has traditionally been consumed, could serve as a cheaper source of essential nutrients than over-the-counter products or prescription drugs.

Although the lipid content of adult *S. interrupta* is low, it is with 6.63%, however, higher than the amount found in most conventional foods like beef (3.6%) and chicken (6%) widely consumed in West Africa [26]. The values for the degree of lipid saturation show that the studied species contains saturated fatty acids at a level of 44.24 ± 0.03%. Saturated fatty acids are excellent sources of energy, but their excessive consumption may increase the risk of cardiovascular disease and promote thrombosis formation [53]. However, stearic acid (present in *S. interrupta* to approximately 9%) has been credited with a protective function regarding cholesterol [54].

The main monounsaturated fatty acid present in *S. interrupta* is oleic acid (48.69 ± 0.02%). The high oleic acid content is a nutritional advantage, as lipids rich in unsaturated fatty acids are beneficial for human consumption [55]. The apparent lack of palmitoleic fatty acid C16:1 (a hexadecenoic acid) is a little surprising, as it has been found in many insects to around 2.5%. However, the insect’s food and larval period undoubtedly play a role in fatty acid composition and abundance. That in *S. interrupta* the amount of unsaturated fats is 10% higher than that of saturated fats is noteworthy, because even in chicken meat, saturated fats are equally or even slightly more abundant than unsaturated fats [56].

Polyunsaturated fatty acids found in the lipids of *S. interrupta* are linoleic acid (3.84 ± 0.00%) and α-linolenic acid (0.96 ± 0.01%). The level of polyunsaturation in the studied insect is low (3.84 ± 0.09%), since the polyunsaturated fatty acids content is below 15% of total fatty acids, as in several other species [33,36]. *S. interrupta* contains both essential fatty acids (linoleic acid and α-linolenic acid), which are the precursors of the omega-6 and omega-3 fatty acid families. The studied species has an omega-6/omega-3 ratio of less than 5, which highlights the nutritional quality of this insect’s fat, as such a ratio is associated with a reduced risk of cardiovascular disease [57]. Linoleic and α-linolenic acids are essential for the proper functioning of the human body. Linoleic acid is incorporated into membrane phospholipids, contributing to membrane fluidity, flexibility, and cellular signaling processes [58], while α-linolenic acid plays specific roles in brain development and the physiology of the nervous system [59].

The fibre content of *S. interrupta* is higher than that of conventional meat products [26], although in insects their exoskeleton is included in the determination of the total amount of fibre present. Nonetheless, the buprestid species investigated by us supports healthy digestion for individuals of all ages since it is known that dietary fibre, irrespective of whether derived from insect chitin or not, can improve gut health [60]. In addition, fibre has a positive effect on enhancing satiety, thereby reducing the risk of overeating and obesity [61]. Finally, it must not be dismissed that a certain proportion of humans possess in their gastric juice enzymes that can digest insect chitin [62].

## 5. Conclusions

Three species of buprestids were identified as being consumed in Togo during this study. This work, which constituted a first assessment aimed at identifying the buprestid species consumed in Togo, should be followed by more intensive studies within the ecosystems of the study area, as this traditional knowledge is disappearing among younger generations. The study showed that the age of the consumer significantly influences buprestid consumption practices. This reflects a generational shift, marked by dietary transition and the gradual erosion of traditional practices. The decline in buprestid consumption among young people is primarily explained by ecological scarcity, combined with changes in dietary habits and cultural marginalization, creating an environment that is unfavourable to the continuation of this tradition. To reverse this trend, concerted efforts are needed, focusing on cultural valorization, ecological protection of species, and raising awareness among youth about traditional food practices. *S. interrupta*, the species whose chemical composition was analysed, contains all essential amino acids required by the human body. It provides lipids rich in unsaturated and essential fatty acids, as well as minerals and vitamins vital to human health. Although chitin can act as an antinutrient and negatively affect the uptake of minerals, e.g., iron [63] and some other antinutrients, such as tannin and phytic acid may also exist in small amounts (as they do in other insects, e.g., *Odontotermes* sp. and *O. smaragdina* [33]), they can be ignored as the balance of essential amino acids, fatty acids, and variety of minerals in *S. interrupta* renders this species highly nutritious. Moreover, as the authors Mwangi et al. [63] of their 2022 paper conclude: “Our study was of an exploratory nature and therefore needs further confirmation by future studies”. The quantity and quality of the nutrients in *S. interrupta* suggest that it could serve as a valuable resource for applications in the agri-food industry.

## Figures and Tables

**Table 1:** Taxonomy of consumed buprestid species.

Genera	Scientific Names of Species
*Sternocera*	*Sternocera castanea* (Olivier, 1790)
	*Sternocera interrupta* (Olivier, 1790)
*Steraspis*	*Steraspis squamosa* (Klug, 1829)

**Table 2:** Number (percentage) of buprestid consumers by age (y) based on 90 people surveyed in each ethnic group.

Ethnic Group	Generations (Ages in Years)			Total (%)
	5–30 y (%)	30–60 y (%)	60–85 y (%)	
Anoufo	9 (10.00%)	14 (15.56%)	22 (24.44%)	45(50.00%)
Bassar	4 (4.44%)	14 (15.56%)	20 (22.22%)	38(42.22%)
Gourma	9 (10.00%)	16 (17.78%)	22 (24.44%)	47(52.22%)
Komkomba	3 (3.33%)	15 (16.67%)	24 (26.67%)	42(46.67%)
Lamba	7 (7.78%)	15 (16.67%)	21 (23.33%)	43(47.78%)
Moba	8 (8.89%)	19 (21.11%)	22 (24.44%)	49(54.44%)
Ngam-gam	7 (7.78%)	16 (17.78%)	22 (24.44%)	45(50.00%)
Total	47 (7.46%)	109 (17.30%)	153 (24.29%)	309(49.05%)

**Table 3:** Proximate composition (% dry weight) and energy value (kJ/100 g) of *S. interrrupta*.

Parameters Analysed	Proximate Composition and Energy Value
Ash	9.48 ± 0.45
Protein	58.02 ± 0.18
Lipids (Fat)	6.63 ± 0.28
Fibre	12.81 ± 0.49
Nitrogen free extract	13.06 ± 0.74
Energy	446.47 ± 5.57

**Table 4:** Mineral composition (mg/100 g dry weight) of *S. interrrupta*, compared with recommended dietary intakes [35].

Mineral	Average Content	RDI [35] Adults	% of RDI per 100 g
Calcium	198.09 ± 1.82	1000 mg/day	19.8%
Magnesium	98.82± 0.06	420 mg/day (men), 360 mg/day (women)	23.5% (men), 27.5% (women)
Phosphorus	183.28 ± 2.76	700 mg/day	26.2%
Potassium	635.56 ± 1.48	3500 mg/day	18.2%
Sodium	22.11 ± 0.17	<2000 mg/day (maximum)	1.1%
Iron	11.86 ± 0.02	11 mg/day (men), 16 mg/day (women)	107.8% (men), 74.1% (women)
Manganese	5.71 ± 0.07	2 mg/day	285.5%
Copper	2.84 ± 0.11	1.6 mg/day (men), 1.3 mg/day (women)	177.5% (men), 218.5% (women)
Zinc	11.49 ± 0.17	11 mg/day (men), 8 mg/day (women)	104.5% (men), 143.6% (women)

**Table 5:** Sodium/potassium, calcium/phosphorus and calcium/magnesium ratios for *S. interrrupta*.

Ratios	Values
Sodium/Potassium	0.03
Calcium/Phosphorus	1.08
Calcium/Magnesium	2.00

**Table 6:** Average vitamin contents of *S. interrrupta* (mg/100 g dry weight), compared to the RDI.

Vitamin	Average Content (mg/100 g)	RDI [35]	% of RDI per 100 g
Retinol (A)	0.7 mg	0.7 mg	2.9%
Thiamin (B1)	1.2 mg	1.2 mg	106.7%
Riboflavin (B2)	1.2 mg	1.2 mg	119.2%
Niacin (B3)	14 mg	14 mg	51.0%
Tocopherol (E)	12 mg	12 mg	34.2%

**Table 7:** Amino acid profile (g/100 g) of *S. interrrupta*.

Amino Acids	Values (±SD)
Isoleucine	1.99± 0.04
Leucine	3.81 ± 0.09
Lysine	1.69 ± 0.01
Methionine	1.06 ± 0.03
Phenylalanine	1.21 ±0.03
Tryptophan	0.48 ±0.05
Threonine	1.17 ± 0.00
Valine	2.51 ± 0.01
Histidine	1.86 ± 0.05
Cysteine	0.86 ± 0.01
Arginine	1.37 ± 0.02
Alanine	2.98 ± 0.04
Aspartic acid	3.20 ± 0.01
Glutamic acid	4.59 ± 0.01
Glycine	4.63 ± 0.14
Proline	2.96 ± 0.05
Serine	1.46 ± 0.00
Tyrosine	6.55 ± 0.29

**Table 8:** Index values for amino acids of *S. interrrupta* by comparison with WHO/FAO/UN reference values.

Amino Acids	Reference WHO/FAO/UN [31]	Index Values for *S. interrrupta*
Threonine	0.9	130
Valine	1.3	193.07
Methionine + Cysteine	1.7	129.94
Isoleucine	1.3	153.07
Leucine	1.9	200.52
Tyrosine +Phenylalanine	1.9	408.42
Histidine	1.6	116.25
Lysine	1.6	105.63
* Tryptophan	0.5	96
Amino acid index	100	96

* Limiting amino acid: Tryptophan.

**Table 9:** Fatty acid profile (g/100 g of total fatty acids) of *S. interrupta* lipids.

Fatty Acids	Percentage (± SD)
Lauric acid (C12:0)	1.03 ± 0.02
Isopalmitic acid (C16:0)	34.09 ± 0.01
Stearic acid(C18:0)	9.12 ± 0.02
**Saturated fatty acids**	**44.24 ± 0.03**
Oleic acid (Cl8:1)	48.69 ± 0.02
Linoleic acid (C18:2n 6)	3.84 ± 0.00
α-linolenic acid (C18:3)	0.96± 0.01
**Unsaturated fatty acids**	**54.45 ± 0.83**
Omega-6/Omega-3	4

## Data Availability

The original contributions presented in this study are included in the article/Supplementary Materials. Further inquiries can be directed to the corresponding author.

## Supplementary Materials

The following are available online at https://www.mdpi.com/article/10.3390/insects17030320/s1, Table S1: Ethnoentomological data survey sheet on edible jewel beetles in Togo.

## Abbreviations

The following abbreviations are used in this manuscript:

AFNOR	French Association for Standardization
ANSES	French Agency for Food, Environmental and Occupational Health and Safety
AOAC	Association of Official Analytical Chemists
AOCS	American Oil Chemists’ Society
FAO	Food and Agriculture Organization of the United Nations
INFOODS	International Network of Food Data Systems
RDI	Recommended Daily Intakes
UN	United Nations
WHO	World Health Organization
