# Supplementary material for: Buprestid Beetles of Togo: Ecological, Sociocultural, and Nutritional Impacts of a High Quality Food Source
Source: Insects. 2026 Mar 16;17(3):320. doi: 10.3390/insects17030320 (PMC13027031; doi:10.3390/insects17030320)
Supplement: Supplementary file 1 [file insects-17-00320-s001.zip › insects-4186514-supplementary.pdf]

Table S1: Ethnoentomological Data Survey Form for Edible Jewel Beetles in Togo

Ethnic Group Name	Age of Respondent	Sex of Respondent	Name of Locality	Date

A) Information on all edible jewel beetles in the area

No.	Local Name of Consumed Jewel Beetle	French Name of Consumed Jewel Beetle	Description of Jewel Beetle
1			
...			
5			

No.	Local Name(s) of Host Plant(s)	French Name(s) of Host Plant(s)	Description of Consumed Jewel Beetle Habitat
1			
...			
5			

No.	Stage(s) and Local Name(s) of Consumed Jewel Beetle Stage(s)	Period (months) of Consumed Stage(s)	Current abundance of the consumed stage(s) in the environment (+: low, ++: medium, +++: high)	Abundance in the past (10 years) of the consumed stage(s) in the environment (+: low, ++: medium, +++: high)
1				
...				
5				

B) Socio-cultural data and marketing

No.	Collection basis (community or individual)	Age group of the jewel beetle harvester			Sex of the harvester
		Young	Adults	Elderly	
1					
...					
5					

No.	Harvesting equipment	Harvesting technique	Presence of toxin in the jewel beetle
1			
...			
5			

No.	Culinary technique	Method of consumption	Consumption period	Method of preservation
1				
...				
5				

No.	Other uses (medicinal or stimulant)	Taboos associated with the consumption of each jewel beetle	Marketing of the species (Yes/No)
1			
...			
5			

D) Factors contributing to the decline in jewel beetle consumption

No.	Reduced availability	Marginalization	Dietary substitution	Urbanization
1				
...				
5				

No.	Deculturation	Change in taste	Other
1			
...			
5			
